# Complex Assessment of Metabolic Effectiveness of Insulin Pump Therapy in Patients with Type 2 Diabetes Beyond HbA1c Reduction

**DOI:** 10.1089/dia.2017.0283

**Published:** 2018-02-01

**Authors:** Rudolf Chlup, Sarah Runzis, Javier Castaneda, Scott W. Lee, Xuan Nguyen, Ohad Cohen

**Affiliations:** ^1^ Department of Physiology, Faculty of Medicine and Dentistry, Palacký University, Olomouc, Czech Republic.; ^2^ IInd Department of Medicine, Teaching Hospital, Olomouc, Czech Republic.; ^3^ Department of Diabetes Moravsky Beroun, Institute Paseka, Paseka, Czech Republic.; ^4^ Medtronic, Sarl, Tolochenaz, Switzerland.; ^5^ Medtronic, Bakken Research Center, Maastricht, The Netherlands.; ^6^ Medtronic, Northridge, California.; ^7^ Medtronic, Tolochenaz, Switzerland.

**Keywords:** Insulin pump, Type 2 diabetes, Insulin aspart, HbA1c, Body mass, Self-monitoring.

## Abstract

***Background:*** This prospective single-center study recruited insulin-resistant continuous subcutaneous insulin infusion (CSII) therapy-naive patients with type 2 diabetes (T2D) using insulin analog-based multiple daily injections (MDI) therapy and metformin.

***Methods:*** A total of 23 individuals with T2D (70% male), aged a mean ± standard deviation 57.2 ± 8.03 years, with body mass index of 36.2 ± 7.02 kg/m^2^, diabetes duration of 13.3 ± 4.64 years, and HbA1c of 10.0% ± 1.05% were randomly assigned to a CSII arm or an MDI continuation arm to explore glucose control, weight loss, total daily insulin dose (TDD), and insulin resistance. Insulin dosing was optimized over a 2-month run-in period.

***Results:*** At 6 months, patients assigned to the CSII arm achieved a significant mean HbA1c reduction of −0.9% (95% confidence interval [CI] = −1.6, −0.1), while reducing their TDD by −29.8 ± 28.41 U/day (33% of baseline [92.1 ± 20.35 U/day]) and achieving body mass (BM) reduction of −0.8 ± 5.61 kg (0.98% of baseline [104.8 ± 16.15 kg]). MDI patients demonstrated a nonsignificant HbA1c reduction of −0.3% (95% CI = −0.8, 0.1) with a TDD reduction of 5% from baseline (99.0 ± 25.25 U/day to 94.3 ± 21.25 U/day), and a BM reduction of −1.0 ± 2.03 kg (0.99% of baseline [108.9 ± 20.55 kg]). After 6 months, the MDI arm crossed over to CSII therapy. At 12 months, patients continuing CSII demonstrated an additional mean 0.7% HbA1c reduction with 54.6% achieving HbA1c<8%. The final TDD reduction was −9.7 U/day in comparison to baseline; BM increased by 1.1 ± 6.5 kg from baseline. The MDI patients that crossed to CSII showed an HbA1c reduction of −0.5% ± 1.04%, HbA1c response rate of 27.3%, a TDD reduction of −17.4 ± 21.06 U/day, and a BM reduction of −0.3 ± 3.39 kg. Diabetic ketoacidosis or severe hypoglycemia did not occur in either arm.

***Conclusion:*** CSII therapy safely and significantly improved metabolic control with less insulin usage, with no sustainable reduction of BM, blood pressure, and lipid profile, in insulin-resistant T2D patients. Treatment adherence and satisfaction in these patients were excellent.

## Introduction

Several clinical studies demonstrated improved metabolic control in people with type 2 diabetes (T2D) using preprandial supplementary or complementary multiple daily injections (MDI) therapy.^1–8^ A less physiological approach that appears to be effective in reducing HbA1c is titration with a single dose of long-lasting insulin or a combined basal-bolus concept.^9,10^ It is now well established that continuous subcutaneous insulin infusion (CSII) therapy can further decrease HbA1c concentrations when compared to MDI.^11–16^ However, the primary objective in these studies was to reduce HbA1c by adjusting the daily insulin dose, with less attention paid to other parameters. In contrast to these compelling works, there were randomized trials of CSII versus MDI, which reported no advantages regarding the CSII therapy.^17,18^ In addition, there have been nonrandomized studies with positive results with CSII therapy.^19–21^

Taking into consideration potential harmful effects of hyperinsulinemia in insulin-resistant diabetes patients,^22–26^ the following study aimed to not only improve glycemia, but also evaluate other relevant metabolic indices. Specifically, glucose control, total daily insulin dose (TDD), body mass (BM), insulin resistance, blood pressure (BP), serum triacylglycerols (TAG), low-density lipoprotein (LDL), high-density lipoprotein (HDL), and diabetes treatment satisfaction were examined at 6 and 12 months post-CSII-start in patients with T2D.

## Methods

### Study design

This prospective single-center, randomized study recruited 36 insulin-resistant, C-peptide-positive, glutamic acid decarboxylase antibodies (GAD Ab)-negative, and CSII-naive patients with T2D (eight screen failures). Insulin resistance was empirically defined as a required insulin dose between 0.5 and 1.8 U/kg of BM per day. These patients who were on insulin analog-based MDI therapy and metformin were recommended from eight regional diabetes departments. Exclusion criteria included active malignancy; acute cardiovascular disorders; acute renal failure; treatment with sulphonylurea, and/or corticosteroids, and/or incretin analogs; pregnancy; and/or poor compliance. Informed consent approved by the local Ethics Committee was obtained from all patients. After enrollment, 28 patients were required to undergo a 2-month run-in period. Insulin dosing was optimized on MDI with insulin analogs and metformin dose was increased to 3000 mg/day.

Following the run-in period, patients were randomized into two groups: a CSII arm or an MDI continuation arm. After 6 months, patients receiving MDI therapy had the option to cross over to CSII therapy: both arms were followed up during 6 additional months, making a total study period of 12 months. CSII was performed using MiniMed™ Veo™ insulin pumps and respective consumables delivering insulin aspart. Before the start of CSII therapy, education on insulin pump therapy was carried out by trained professionals over approximately five sessions. There were 10 scheduled visits in each arm. At Visit 1, patients were randomized into the CSII or MDI arm. At 3 months (Visit 4 or 9), the TDD was incrementally increased in steps if no HbA1c improvements, compared to baseline, were observed.

Cross over from the MDI to CSII arm occurred at 6 months (Visit 5), and a final visit (Visit 10) occurred at 12 months. In both arms, at the CSII start (Visit 1 or Visit 5, respectively), the TDD was reduced by 10%–50% in order not to exceed 80 U/day, without considering the actual HbA1c level.

### Self-monitoring and laboratory investigations

The mean frequency of self-monitoring on a personal glucose meter (CONTOUR^®^LINK; Ascensia Diabetes Care, Parsippany, NJ) varied in both arms between 3.4 and 5.4 measurements per day.

In addition, glycemia was evaluated with continuous glucose monitoring (CGM) using the Medtronic iPro™ Professional CGM system, in three blinded 6-day periods (baseline, end of the study phase, end of the continuation phase). Sensors were inserted before Visit 1, Visit 5, and Visit 10.

Laboratory investigations were performed at Covance Laboratories (Switzerland) in which HbA1c was analyzed using a Diabetes Control and Complications-standard assay and given in National Glycohemoglobin Standardization Program (NGSP) units [%] based on NGSP. To convert these NGSP values (reference range 4.2%–6.0%) to the recent International Federation of Clinical Chemistry (IFCC) values (reference range 22–42 mmol/mol), the following equation was used: IFCC HbA1c (mmol/mol) = (NGSP HbA1c [%]–2.15)/0.0915 and rounded (no decimal points).

### Statistical evaluation

The primary efficacy endpoint was the reduction in HbA1c from baseline to 6 months, which was analyzed with a two-sided, two-sample, *t*-test including available measurements. After the initial 6 months of the study phase, patients continued an additional 6 months of CSII therapy. Data at the 12-month visit were pooled to assess 1-year change from baseline using a paired *t*-test. Descriptive statistics for continuous variables included mean, standard deviation, and 95% confidence interval (CI) limits. All *P*-values were two-sided, and those below 0.05 were considered statistically significant. In addition, the Diabetes Treatment Satisfaction Questionnaire (DTSQ) scores^27^ for the status version (DTSQs) and the change version (DTSQc) were analyzed to look at changes in satisfaction over time: from screening and baseline (i.e., randomization) to 6 months of therapy (Visit 5) and 12 months of therapy (Visit 10). The DTSQc was collected only at Visit 5 and Visit 10.

## Results

### Run-in period and randomization

Following the run-in period, 23 patients (70% male) were randomized into two groups: a CSII arm (*n* = 11) or an MDI continuation arm (*n* = 12). These patients had persistent HbA1c ≥8% [64 mmol/mol], mean ± standard deviation age of 57.2 ± 8.0 years, body mass index (BMI) of 36.2 ± 7.0 kg/m^2^, BM of 106.9 ± 18.3 kg, diabetes duration of 13.3 ± 4.7 years, and HbA1c of 9.5% ± 0.96% [80 mmol/mol]). The other remaining 5 of 28 patients (18%) were not randomized due to HbA1c <8% [64 mmol/mol] at the end of the run-in period. After 6 months, all patients from the MDI arm (except one) crossed over to CSII therapy, and all were followed up for an additional 6 months. In total, 11 completed 12 months of CSII (CSII/CSII arm) and 11 completed 6 months of CSII, after the initial 6 months of MDI (MDI/CSII arm).

### Follow-up visits

Patients assigned to the CSII arm achieved a significant mean HbA1c reduction of 0.9% ± 1.1% [10 ± 12 mmol/mol] (95% CI = −1.6 [−17], −0.1 [−1]; *P* = 0.0312), while reducing their TDD by 29.8 ± 28.41 U/d (33% of baseline 92.1 ± 20.35 U/d), achieving a BMI reduction of −0.3 ± 1.94 kg/m^2^ (95% CI = −1.61, 0.99) (0.86% of baseline 36.2 ± 4.25 kg/m^2^), and a BM reduction of −0.78 ± 5.61 kg (95% CI = −4.55, 2.99) (0.74% of baseline 104.8 ± 16.15 kg).

Patients on MDI demonstrated a nonsignificant HbA1c reduction of 0.3% [3 mmol/mol] (95% CI = −0.8 [−9], 0.1 [1]) with a TDD reduction of 0.4 U/d from baseline (99.0 ± 25.25 U/d), a BMI reduction of −0.31 ± 0.70 kg/m^2^ (95% CI = −0.78, 0.16) (0.9% of baseline 36.2 ± 9.07 kg/m^2^), and a BM reduction of −1.0 ± 2.03 kg (95% CI = −2.36, 0.36) (0.91% of baseline 108.9 ± 20.55 kg). The between-group difference in 6-month HbA1c reduction was −0.53% ± 0.9% [1 ± 10 mmol/mol] in favor of the CSII arm, but it was not significant (*P* = 0.20).

At 12 months, data from both arms (*N* = 11 continuing CSII from the randomization visit for 12 months, and *N* = 11 following crossover from MDI to CSII at Visit 5 for 6 months) demonstrated a mean 1.2% ± 0.87% [13 ± 10 mmol/mol] HbA1c reduction from baseline (9.5% [80 mmol/mol]) (*P* < 0.0001) with 41% of patients achieving HbA1c<8% (64 mmol/mol); a mean TDD reduction from baseline (95.7 ± 22.75 U/d) of 13.7 ± 29.7 U/d (i.e., 0.14 U/d/kg of BM). No significant change versus baseline was noted in terms of BM (107.0 kg vs. 107.1 kg). In addition, and relative to baseline, results showed systolic BP of 140.7 torr versus 139.6 torr; diastolic BP of 83.3 torr versus 78.3 torr; HDL of 1.2 mmol/L versus 1.2 mmol/L; LDL of 2.2 mmol/L versus 2.3 mmol/L; and TAG of 2.8 mmol/L versus 2.2 mmol/L; with no ketoacidosis or severe hypoglycemia occurring in either arm.

**FIG. 1.**
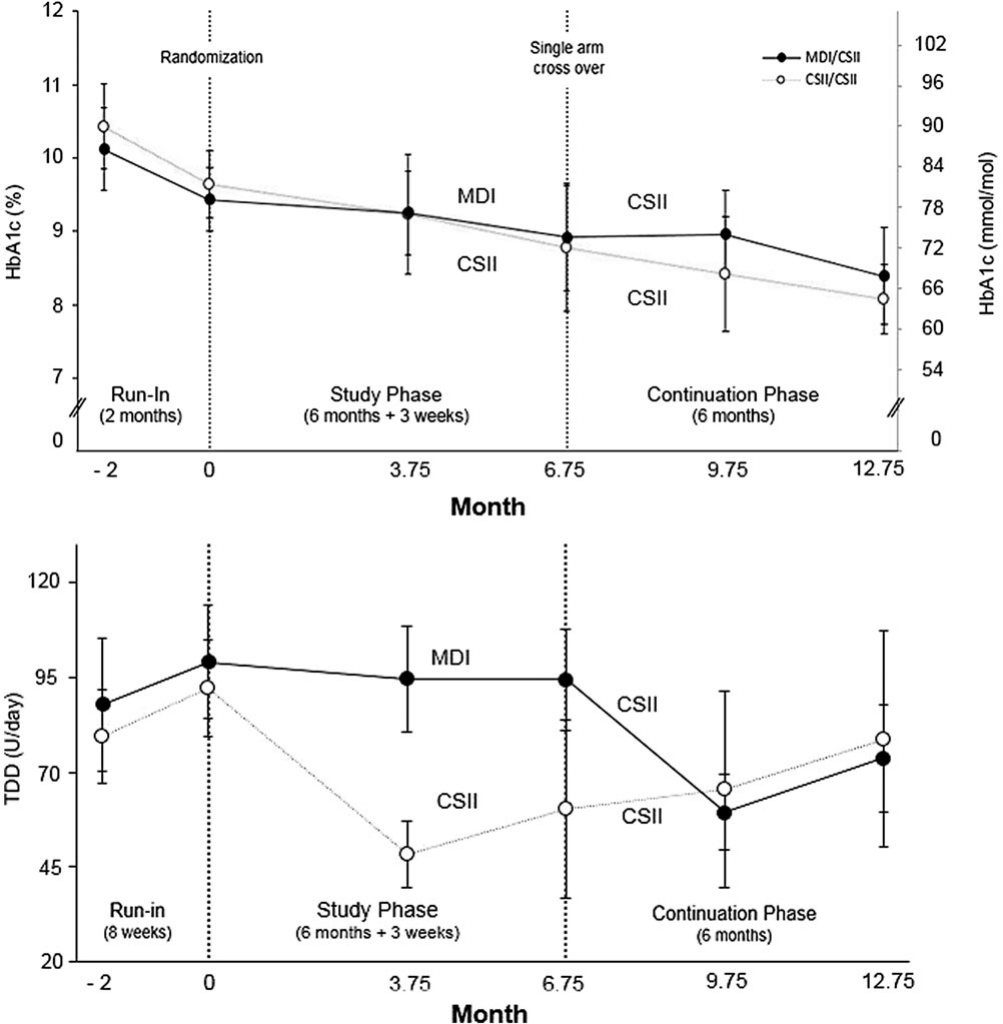
HbA1c and insulin total daily dose during the course of the study. HbA1c and TDD during the course of the study. The HbA1c (top) and TDD (bottom) in the MDI/CSII arm (*N* = 11, closed symbols) and the CSII/CSII arm (*N* = 11, open symbols) are shown. Symbols and bars show the mean and 95% CI, respectively. CI, confidence interval; CSII, continuous subcutaneous insulin infusion; MDI, multiple daily injections; TDD, total daily insulin dose.

**FIG. 2.**
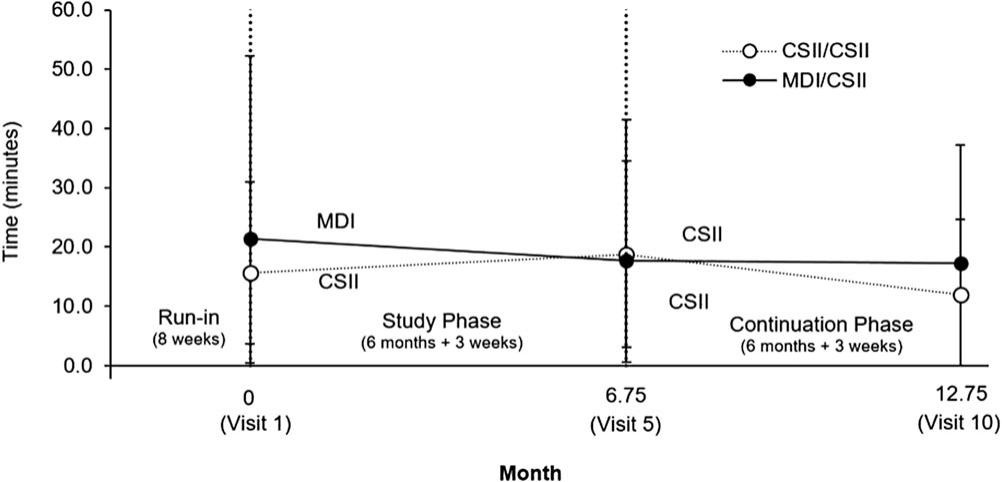
Mean daily hypoglycemia exposure during the study. The mean time (in minutes) of hypoglycemia (≤70 mg/dL, ≤3.9 mmol/L) exposure per day is shown for the MDI/CSII arm (*N* = 11, closed symbols) and the CSII/CSII arm (*N* = 10, open symbols) at baseline (Visit 1), 6 months (Visit 5), and 12 months (Visit 10, end of study). While hypoglycemia exposure was reduced in the MDI arm that transitioned to CSII therapy by end of study (Visit 10), this was NS when compared to baseline. For the CSII/CSII arm, hypoglycemia exposure was increased from Visit 5 to end of study (NS, compared to baseline). Symbols and bars show the mean and 95% CI, respectively. NS, not significant.

The course of HbA1c and TDD in the individual study arms are displayed in Figure 1. The mean time spent per day in hypoglycemia (≤70 mg/dL [≤3.9 mmol/L]) at baseline (Visit 1), Visit 5, and Visit 10 is also shown in Figure 2. Compared to baseline, there was no significant change in the percentage of time spent in hypoglycemia at the 6-month visit (MDI/CSII 95% CI = [−2.5%, 1.5%], CSII/CSII 95% CI = [−2.5%, 3.5%]). Likewise, no significant change was observed when comparing hypoglycemia exposure between baseline and the 12-month visit. Compared to baseline, there was no significant reduction in the mean amplitude of glycemic excursions for the MDI/CSII arm (−3.0 [33.6] and −8.7 [29.0]) or the CSII/CSII arm (−12.9 [20.6] and −15.1 [27.2]) at the 6-month and 12-month visits, respectively. The standard deviation of sensor glucose values (i.e., variability) decreased at the 6- and 12-month visits for the CSII/CSII arm (−4.7 ± 7.9 mg/dL[−0.26 ± 0.44 mmol/L] and −4.8 ± 7.6 mg/dL [0.27 ± 0.42 mmol/L], respectively), but was not statistically significant. Compared to baseline, neither the increased standard deviation of sensor glucose values for the MDI/CSII arm at 6 months (5.8 ± 2.0 mg/dL [0.32 ± 0.11 mmol/L]), nor the decreased standard deviation of sensor glucose at 12 months (−3.8 ± 9.4 mg/dL [−0.21 ± 0.52 mmol/L]) was significant.

### DTSQ scores over time

Tables 1 and 2 show the results of DTSQs and DTSQc questionnaires, respectively, during the study and changes in scores over time, for both the CSII/CSII and MDI/CSII arms. Relative to baseline, there was a small increase in the DTSQs for the CSII/CSII arm at 6 months that was minimally increased by the end of the study. For the MDI/CSII arm, the DTSQs were minimally reduced at 6 months relative to baseline, but comparable to that of the CSII/CSII arm by 12 months. Patients within both arms reported similar DTSQc scores at 6 and 12 months, with a comparable increase in change between the visits.

**Table 1.** Change in the Diabetes Treatment Satisfaction Questionnaire (Status) Score

		*Run-in*						
*Treatment arm*	*Category*	*Screening*	*Baseline*	*6 months (Visit 5)*	*Study phase 6 months change from baseline*	*Continuation phase 12 months (Visit 10)*	*12 months change from 6 months*	*12 months change from baseline*
CSII/CSII	Number of subjects	11	11	11	11	11	11	11
	Mean (SD)	25.9 (5.11)	30.4 (3.50)	31.6 (2.66)	1.3 (3.55)	32.9 (3.05)	1.3 (1.74)	2.6 (3.21)
	Median	26.0	30.0	31.0	0.0	34.0	1.0	1.0
	Minimum, maximum	18.0, 35.0	25.0, 36.0	28.0, 36.0	−3.0, 8.0	27.0, 36.0	−1.0, 4.0	−1.0, 9.0
	95% confidence interval	22.5, 29.3	28.0, 32.7	29.9, 33.4	−1.1, 3.7	30.9, 35.0	0.1, 2.4	0.4, 4.7
	Interquartile range (25%, 75%)	21.0, 29.0	27.0, 34.0	30.0, 34.0	−2.0, 3.0	31.0, 36.0	0.0, 3.0	0.0, 5.0
MDI/CSII	Number of subjects	12	12	11	11	11	11	11
	Mean (SD)	24.8 (5.18)	30.3 (4.99)	30.1 (5.86)	−0.5 (4.59)	32.9 (3.83)	2.8 (4.92)	2.4 (2.80)
	Median	24.0	31.0	33.0	0.0	34.0	2.0	3.0
	Minimum, maximum	18.0, 34.0	19.0, 36.0	19.0, 35.0	−10.0, 7.0	23.0, 36.0	−3.0, 12.0	−2.0, 8.0
	95% confidence interval	21.5, 28.1	27.1, 33.4	26.2, 34.0	−3.5, 2.6	30.3, 35.5	−0.5, 6.1	0.5, 4.2
	Interquartile range (25%, 75%)	21.0, 29.0	28.5, 34.0	26.0, 34.0	−4.0, 2.0	31.0, 36.0	−2.0, 7.0	0.0, 4.0

CSII, continuous subcutaneous insulin infusion; MDI, multiple daily injections; SD, standard deviation.

**Table 2.** Change in the Diabetes Treatment Satisfaction Questionnaire (Change) Score

*Treatment arm*	*Category*	*Study phase 6 months (Visit 5)*	*Continuation phase 12 months (Visit 10)*	*12 months change from 6 months*
CSII/CSII	Number of subjects	11	11	11
	Mean (SD)	13.1 (3.53)	16.1 (2.26)	3.0 (3.63)
	Median	12.0	17.0	2.0
	Minimum, maximum	6.0, 18.0	12.0, 18.0	−1.0, 11.0
	95% confidence interval	10.7, 15.5	14.6, 17.6	0.6, 5.4
	Interquartile range (25%, 75%)	11.0, 16.0	13.0, 18.0	0.0, 5.0
MDI/CSII	Number of subjects	11	11	11
	Mean (SD)	13.6 (5.05)	16.3 (1.95)	2.6 (4.25)
	Median	16.0	17.0	1.0
	Minimum, maximum	1.0, 18.0	13.0, 18.0	−2.0, 13.0
	95% confidence interval	10.2, 17.0	15.0, 17.6	−0.2, 5.5
	Interquartile range (25%, 75%)	11.0, 17.0	14.0, 18.0	0.0, 3.0

The Diabetes Treatment Satisfaction Questionnaire was only collected at Visit 5 and Visit 10.

### Adverse events

There was a total of 31 various adverse events registered in 8 of 22 patients when using CSII during the study and continuation phase. Only one of the events (i.e., weakness, slight headache, sweating, and tremor followed by short unconsciousness with spontaneous recovery), which appeared at home without medical assistance or glycemia monitoring, was deemed related to diabetes.

## Discussion

The purpose of this study was to improve the HbA1c and BM in obese insulin-resistant T2D patients using the smallest TDD of rapid-acting insulin analog and maximum dose of metformin. The diagnosis of T2D was based on clinical assessment of the referring diabetes specialist supported by prestudy findings of normal or increased C-peptide concentrations and GAD Ab within the reference range. Insulin therapy in T2D (previously described as noninsulin dependent diabetes) relates to very old references.^1–9^ Some authors preferred the physiological approach with MDI and complemented the missing peak of postprandial endogenous insulin secretion (using small subcutaneous doses of short-acting insulin or insulin analog before each meal, adopted according to intensive self-monitoring).^1–8^ Other authors recommended a more comfortable regimen using once-daily injection of long-lasting insulin titrated according to a single value of fasting glycemia. The latter approach often resulted in increased BM and increased frequency of hypoglycemia.^9^

Regarding CSII therapy in patients with T2D; between 2003 and 2005, there were two randomized trials of CSII versus MDI, which demonstrated no advantages with CSII therapy. In contrast, several nonrandomized pilot studies, between 2010 and 2011, reported optimistic results.^11,12,19–21^ As late as 2016, the outcomes of the randomized controlled OpT2mise study^13–16^ have demonstrated significant superiority of CSII therapy over MDI therapy with HbA1c as primary endpoint.

In this study, over the course of 3 months with CSII therapy, the TDD in the CSII/CSII arm was reduced from 92.1 U/d at baseline to 48.5 U/d by Visit 4. In contrast, the TDD in the MDI/CSII arm was reduced from 94.3 U/d at Visit 5 to 59.4 U/d by Visit 9 (i.e., as late as after crossing over to CSII) and showed no significant increase of HbA1c. Thus, CSII was more effective than MDI (perhaps due to the more physiological mode of basal insulin administration with insulin pump infusion therapy). When the TDD was increased in the CSII/CSII arm from 48.5 U/d (Visit 4) to 78.8 U/d (Visit 10), or in the MDI/CSII arm from 59.4 U/d (Visit 9) to 73.7 U/d (Visit 10), there was a significant HbA1c reduction in the CSII/CSII arm from 9.2% (Visit 4) to 8.1% (Visit 10) (77–65 mmol/mol) and in the MDI/CSII arm from 9.0% (Visit 9) to 8.4% (Visit 10) (75–68 mmol/mol), respectively (Fig. 1), without any significant change in the frequency of hypoglycemia (Fig. 2). Our findings correspond to the meta-analysis of randomized trials.^28^ Thus, reducing a previously high TDD at CSII start (without considering actual glycemia or HbA1c value) appears to be worthy of further investigation. In addition, despite significant reduction of HbA1c, it is necessary to bear in mind that only 41% of 22 patients using CSII achieved HbA1c <8% (64 mmol/mol) and no significant change versus baseline was noted in terms of BM (107.0 kg vs. 107.1 kg). Therefore, the potential combination of CSII with recently appearing incretins and/or gliflozins may also become a topic for future research.^29,30^

## Conclusion

The use of CSII therapy in individuals with insulin-resistant T2D is both safe and effective for improving glucose control and reducing insulin usage, although without a sustainable reduction in BM, BP, or lipid profile. While an optimum metabolic balance in most of the patients was not reached, treatment adherence and satisfaction were excellent. All subjects decided to continue using CSII therapy.
